# Characterization of taurine as anti-obesity agent in C. elegans

**DOI:** 10.1186/1423-0127-17-S1-S33

**Published:** 2010-08-24

**Authors:** Hye Min Kim, Chang-Hee Do, Dong Hee Lee

**Affiliations:** 1 Department of Life Sciences, University of Seoul, Dongdaemun-Gu, Seoul, 130-743, Korea; 2 College of Agriculture and Science, Chungnam National University, Yuseong, Daejeon, 305-764, Korea

## Abstract

**Background:**

Taurine plays an important role in reducing physiological stress. Recent studies indicated that taurine may serve as an anti-obesity agent at the cellular level. This study characterizes taurine’s potential anti-obesity function in *C. elegans,* which have become a popular in vivo model for understanding the regulatory basis of lipid biosynthesis and deposition.

**Methods:**

Two strains of *C. elegans* were raised on a normal or high-fat diet: N2 (normal) and RB1600, a mutant in *tub-1* that serves as a tubby homologue and functions parallel to the 3-ketoacyl-CoA thiolase gene (*kat-1)* in regulating lipid accumulation. Taurine’s effect on lipid deposition was characterized according to assays of Sudan black B staining, triglyceride content measurement, food consumption, and mobility comparison.

**Results:**

When N2 was treated with taurine after the culture in the high-fat media, the worms showed lower lipid accumulation in the assays of the Sudan black B staining and the triglyceride quantification. The anti-obesity effect was less evident in the experiment for RB1600. When the amount of taurine was increased for the high-fat-diet-treated N2 strain, fat deposition decreased and mobility increased in a dose-dependent manner. In the food consumption assays, taurine did not cause a significant change in food intake.

**Conclusions:**

Taken together, these results strongly imply that taurine plays an important role in reducing fat deposition by modulating cellular pathways for lipid accumulation and stimulating mobility, but not the pathways for lipid biosynthesis and food intake.

## Background

Taurine, or 2-aminoethanesulfonic acid, is the major component of bile and is present in many types of animal tissues, including in the gall bladder, blood vessels, eyes, and lower intestine. Although taurine is less known as an amino acid, it is commonly used as a functional component of various types of health drinks. It plays a vital role in adjusting physiological pathways in antioxidation and detoxification. Recent studies indicated that taurine deficiency is associated with persistent obesity, which can be prevented by dietary taurine supplementation [1,2]. This study tested taurine’s anti-obesity potential with C. elegans, which are frequently used as a tractable system in current biomedical researches [3,4].

C. elegans is a very useful animal system in unearthing crucial biological mechanisms that are readily applicable to many higher-level organisms, including humans [5,6]. Indeed, many recently discovered biological pathways of C. elegans have been proven analogous to those of mammals, despite its relatively simple body structure [7-9]. Its short generation period of three days enables researchers to perform a drug screen with a time advantage over other test systems, including rodents. Unlike mammals, though, C. elegans lacks leptin-like molecules and their receptor, which participate in regulating fat storage. This makes C. elegans attractive to many researchers who are mass-screening taurine’s potential as an anti-obesity agent, since C. elegans should provide genuine biochemical information on fat storage and utilization that is independent of the hormonal inhibition of food intake. Under certain circumstances, C. elegans serves as a tractable system of studying taurine’s effect on obesity and fat accumulation, and thus, its anti-obesity potential.

Lipid accumulation involves complex processes of lipid biosynthesis, transport, and storage [10-13]. Lipid biosynthesis is mainly controlled by sterol regulatory element-binding proteins (SREBPs). In mammals, stored fat is consumed according to various transcriptional, translational, and post-translational regulation processes, as in mammals. The SREBP cleavage-activating proteins (SCAPs) are believed to play an important role in activating SREBPs before the SREBPs’ transport into the nucleus. The majority of mammalian lipases are homologous to those in C. elegans. In C. elegans, SBP-1, an SREBP homologue, regulates fatty acid homeostasis by modulating the expression of various lipogenic enzymes.

In C. elegans, the gene products of tub-1 are believed to manage lipid accumulation in cooperation with those of kat-1, which govern fatty acid beta-oxidation [14-17]. The tub-1 mutants show an excessive obese phenotype especially under an impaired kat-1 gene. The level of fatty acyl-CoA is very important in controlling the rate of beta-oxidation. Its level is rigorously controlled by carnitine acyltranferase, which transports acyl-CoA and its derivatives into the mitochondrion [18-20]. The enzymatic activity is governed by malonyl-CoA. With increased levels of acetyl-CoA and citrate, however, beta-oxidation decreases in a negative feedback.

This study characterized taurine as an anti-obesity agent in C. elegans that were fed a high-fat diet. The strains of N2, both the normal and RB1600, that mutated in the* tub-1* gene were tested under four classes of experiments. The worms were analyzed using Sudan black B staining, food intake assay, triglyceride measurement, and mobility assessment.

## Methods

### Worm culture and taurine treatment

C. elegans were grown on standard nematode-growth media that contained high or normal content of cholesterol as 25.0 or 12.5 μM, respectively. During each experiment, the worms were grown on the nematode growth media (NGM) that contained *E. coli* OP50 as a food source. The taurine-containing media were prepared by smearing an appropriate amount of taurine stock solution onto the media. The stock of *E. coli* OP50 was prepared by culturing it overnight in an LB medium until concentrated particles were formed. The quantity of OP50 in the media was adjusted depending on the initial worm input and the duration of the worm culture.

### Sudan black B staining

The anti-obesity effect of taurine was studied via microscopy on stained worms. Individual worms were fixed and stained with Sudan black, which stains lipid stores into opaque blue. For the staining, the worms were removed from the culture plates and were starved for 6 h. After they were washed in M9 media, they were fixed with 1% paraformamide in M9. Then they were subjected to three freeze-thaw cycles and dehydrated in 95% ethanol. The staining was performed in 70% ethanol saturated with Sudan black B.

### Lipid content measurement

The lipid contents were quantitated by measuring the triglyceride content of the worms that were treated under taurine or taurine-free conditions. The worms were thoroughly washed in TBS and homogenized and sonicated in a Triglyceride Assay Buffer (TAB) and the triglyceride content was assessed using a BioVision triglyceride assay kit (Mountain view, CA, USA). After brief centrifugation, the samples were diluted to a final volume of 50μl/well. Following the dilution of the samples in two microliters of lipase, the samples were incubated for 20 min at room temperature to convert their triglycerides into glycerol and fatty acid. The optical density of each of the incubated samples was measured at 570 nm for the colorimetric assay in a microplate reader. The calculations were made by subtracting the background value derived from the 0 triglyceride standard from all the sample readings. The triglyceride concentration (nmol/μl) was calculated according to the formula [TC] = Ts (nmol) / Sv (μl) [Ts: amount of triglycerides from the standard curve, and Sv: sample volume (before dilution) in the sample wells].

### Food consumption assay

Worms were treated on taurine or taurine-free media for 12 hours; then, they were subjected either to fast or to copious feeding with OP50 for 6 h on NGM media. Twenty worms of 1-day-old were transferred onto NGM plates covered with lawn of identical amount of OP50 (9 X 10^9^ cfu). The time taken for total intake of the food input was measured and compared among the treatment and genotypes.

### Mobility assay

After the worms’ culture on the tunicamycin-containing media, they were further grown on taurine-containing media. The moving distances of the worms that were treated with various taurine concentrations were visually compared. The difference in the mobility of the taurine-treated and the non-taurine-treated worms was observed in a light box.

## Results

Several experiments were performed to examine the effect of taurine on fat accumulation in the normal and tubby strains of C. elegans. Throughout the various taurine treatments, differences in the lipid accumulation were evident both qualitatively and quantitatively under Sudan black staining, food consumption assay, fatty acid quantification, and mobility comparison analysis.

### Fat accumulation decreased under taurine treatment via Sudan black staining

**Figure 1:**
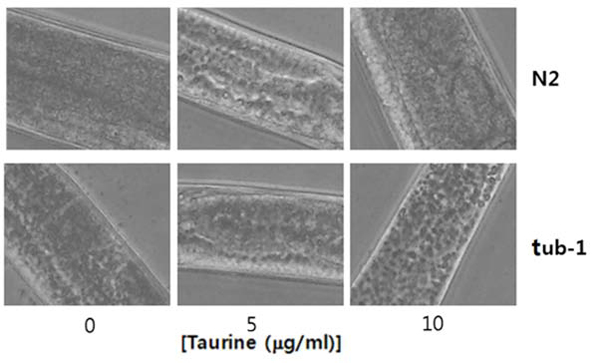
**Sudan black B staining of taurine-treated worms***C. elegans* were treated with taurine for 24 h and its effect on lipid storage was observed by Sudan black B staining. The taurine’s effect on reduction of lipid accumulation was significant in the wild type strain of N2. However, the effect of taurine was minimal or insignificant for the lipid storage mutant,* tub-1*.

The anti-obesity effect of taurine was studied under a microscope. Fig. 1 shows taurine’s effect on lipid storage, as manifested via Sudan black B staining, after the worms were treated for 24 h with two different taurine concentrations. When cultured on a high-fat diet, the worms appeared to accumulate excess triglyceride in separate opaque spots that were highly noticeable in the intestinal and hypodermal tissues. No substantial difference appeared in the fat stores of the worms that were treated with a normal diet (data not shown), but notable differences emerged among the worms that were treated with a high-fat diet followed by taurine treatment. Taurine’s effect of reducing lipid storage was definite in the wild-type strain of N2 (upper panel, Fig. 1), but its effect was minimal or insignificant for *tub-1*, the lipid storage mutant (lower panel).

### The triglyceride level was low in taurine-treated worms

**Figure 2:**
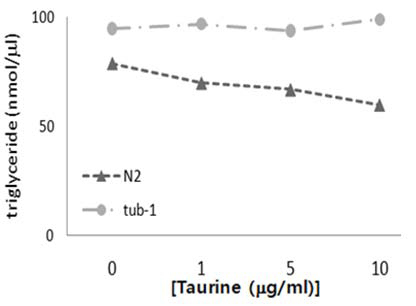
**Triglyceride content measurement** The high fat grown N2 worms positively responded to the taurine treatment in a dose-dependent manner when the triglyceride content was measured as described in Methods. The response of *tub-1*, however, showed no significance compared to N2.

In addition to a visual comparison of the lipid droplet that was formed in the Sudan black B staining experiment, this experiment assessed the total amount of triglycerides in C. elegans. The measurement that was performed in this experiment genuinely referred to the lipid biosynthesis in the worms. The triglyceride content was shown to correspond to those in the experiment and to the dose-dependent anti-obesity effect of taurine (Fig. 2). In the absence of taurine, the normal (N2) strains sensitively responded to the high-fat diet via fat accumulation in a dose-dependent manner. Unlike in the staining experiment, however, the *tub-1* mutant positively responded to taurine by showing a lower triglyceride content under the taurine treatment. The inconsistency between the staining and the quantitation results in the tubby worms implies that taurine may work in the stage of fat biosynthesis rather than in the stage of fat accumulation.

### The taurine treatment did not alter the food consumption assay of the worms

**Figure 3:**
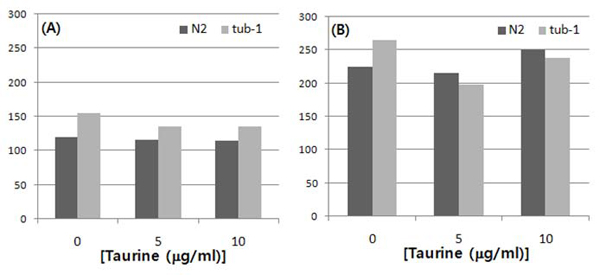
**Food consumption assay** The time taken for the total consumption of OP50 was compared among the taurine treatments. A significant difference was observed between starved (A) and well-fed (B) worms; however, no significance was noticed between the taurine treatments within the strain.  The *x*-axis refers to the time (min) taken for a total consumption of food.

No considerable difference was seen between the worms that were treated with taurine and the worms in the taurine-free conditions. Fig. 3 shows little or no difference between the taurine-treated worms and the non-taurine-treated control, although the starved and the well-fed worms significantly differed in the length of time of their complete food consumption. This observation indicates that taurine may not affect worms’ craving for food, and thus, the potential anti-obesity function of taurine does not stem from the suppression of food ingestion.

### The worms became active under taurine treatment

After the tunicamycin-treated worms were further grown on taurine-containing media, the extents of the worms’ movement after various treatment concentrations were visually compared (Fig. 4). The visual comparison of the worms’ tracks revealed that the taurine-treated worms travelled longer distances than the non-taurine-treated control (Table 1.). The positive effect of taurine on mobility may contribute to the reduction of lipid accumulation in C. elegans. Taurine’s effect was more pronounced when the worms were treated with more taurine.

**Table 1:** Motility comparison assay

	[Taurine]			
***Strain***	0	1	5	10 (μg/ml)
**N2**	d^0^	d^++^	d^+++^	d^+++^
**tub-1**	d^0^	d^+^	d^0^	d^+^

Worms were grown on taurine-containing or free media. The distance of worm’s movement was compared among the taurine treatments. “d^o^” refers to the distance the worms moved on the taurine–free media. “+” denotes an approximate 10% increase in mobility according to the length of path left on the media.

## Discussion

Using C. elegans systems, this study tested taurine’s potential as an anti-obesity agent by helping reduce lipid accumulation in cells. Taurine significantly decreased fat storage depending on its concentration. Without negatively affecting food intake, taurine showed substantially reduced fat accumulation and increased activity, which matched the reduction of lipid storage under its treatment.

Complex cellular mechanisms coordinate the equilibrium between lipid utilization and accumulation [21-23]. Being critical for the utilization of stored lipids, beta-oxidation converts fatty acids to acetyl co-A and requires the release of fatty acids from triacylglycerides by lipases [24-27]. The increased activity of the worms after the taurine treatment strongly suggests that the lesser accumulation of fat was due to the higher level of beta-oxidation from the taurine-enhanced activities of the C. elegans.

One of the findings in this study is that taurine had only a minor anti-obesity effect on the tub-1 mutant, unlike on the normal strain. Unlike the wild type, the tubby mutants are known to store more fat due to their inability to control excessive transport to fat storage tissues or to react properly to metabolic signals from ciliated neurons [28,29]. Thus, taurine’s potential anti-obesity effect is not applicable to the lipid accumulation stage or is independent of the pathways where tub-1 is involved.

The Sudan black B staining and triglyceride assays showed inconsistent results concerning the tub-1 mutants in response to taurine. That is, the Sudan black B staining did not show noticeable sensitivity to taurine application, but the triglyceride content measurements showed a significant difference between the taurine-free and the taurine-treated worms. These inconsistent results strongly indicate that the taurine had a more immediate impact on lipid deposition than on fat synthesis.

As in mammals, C. elegans accumulates fat both from its diet and via de novo biosynthesis, and stores lipids in subcellular compartments. Not enough information is available, however, on how C. elegans acquires, synthesizes, uses, and stores lipids, but the consistent results of this study indicate that taurine diminishes lipid storage by enhancing physical activities, rather than by reducing food uptake. Despite these data, the mechanism behind the subcellular targeting and sequestration that dictate the magnitude and numbers of these deposits remains to be determined.

## Conclusions

In summary, this study showed that taurine affects lipid deposition in a dose-dependent manner. Reduced lipid storage was evident in the wild-type worms but not in the *tub-1* mutants. Unlike tub-1, N2 positively responded to taurine according to its triglyceride content assay. The increased mobility with the taurine treatment may contribute to reduced lipid deposition via enhanced lipid consumption. An additional study is necessary to clarify the potential mechanisms for the fat storage reduction. A new study may be rewarding in finding a definite agent that would modulate tub-1/kat-1 and sbp-1 molecules. Collective treatment of these molecules with taurine is a good way to generally cope with obesity.

## List of abbreviations used

SREBP: sterol regulatory element-binding protein; TAB: triglyceride assay buffer; kat-1: 3-ketoacyl-CoA thiolase-1; NGM: nematode growth media

## Competing interests

The authors declare that they have no competing interests.

## Authors' contributions

HMK maintained C. elegans and carried out the various taurine treatments and data collection. CHD participated in the design of the study and performed the statistical analysis. DHL participated in obtaining worm strains and coordination and helped to draft the manuscript. All authors read and approved the final manuscript.
